# Neuromorphic Implementation of Attractor Dynamics in a Two-Variable Winner-Take-All Circuit with NMDARs: A Simulation Study

**DOI:** 10.3389/fnins.2017.00040

**Published:** 2017-02-07

**Authors:** Hongzhi You, Da-Hui Wang

**Affiliations:** ^1^Key Laboratory for NeuroInformation of Ministry of Education, Center for Information in BioMedicine, School of Life Science and Technology, University of Electronic Science and Technology of ChinaChengdu, China; ^2^School of Systems Science and National Key Laboratory of Cognitive Neuroscience and Learning, Beijing Normal UniversityBeijing, China

**Keywords:** neuromorphic engineering, winner-take-all, attractor dynamics, decision making, working memory, hysteresis

## Abstract

Neural networks configured with winner-take-all (WTA) competition and N-methyl-D-aspartate receptor (NMDAR)-mediated synaptic dynamics are endowed with various dynamic characteristics of attractors underlying many cognitive functions. This paper presents a novel method for neuromorphic implementation of a two-variable WTA circuit with NMDARs aimed at implementing decision-making, working memory and hysteresis in visual perceptions. The method proposed is a dynamical system approach of circuit synthesis based on a biophysically plausible WTA model. Notably, slow and non-linear temporal dynamics of NMDAR-mediated synapses was generated. Circuit simulations in Cadence reproduced ramping neural activities observed in electrophysiological recordings in experiments of decision-making, the sustained activities observed in the prefrontal cortex during working memory, and classical hysteresis behavior during visual discrimination tasks. Furthermore, theoretical analysis of the dynamical system approach illuminated the underlying mechanisms of decision-making, memory capacity and hysteresis loops. The consistence between the circuit simulations and theoretical analysis demonstrated that the WTA circuit with NMDARs was able to capture the attractor dynamics underlying these cognitive functions. Their physical implementations as elementary modules are promising for assembly into integrated neuromorphic cognitive systems.

## Introduction

Winner-take-all (WTA) competition is an important computational principle in the brain, by which neurons can compete for activation. Through configuration of its network parameters, the WTA neural network can achieve various dynamic characteristics of attractors that underlie many brain cognitive functions. For instance, decision-making, a central cognitive process involving selection of an action or an option amongst a set of two or more alternatives, is correlated with neural computation, as reflected by the ramp-up activities in monkeys' lateral intraparietal cortexes (LIP) (Shadlen and Newsome, 2001). If configured with hard WTA, which allows only the neuron or the neural population with the highest activation to stay active, the WTA neural network can implement decision-making (Wang, 2002; Wong and Wang, 2006; You and Wang, 2013). Working memory, the ability to internally maintain information, is manipulated by the sustained activities of cells of the prefrontal cortex during a memory period (Fuster and Alexander, 1971). The WTA neural network also has the capacity to carry out working memory tasks if configured with multi-stable dynamics including the spontaneous attractor and memory attractors (Durstewitz et al., 2000; Wei et al., 2012). Another interesting phenomenon, hysteresis, which shows that the perception change is behind the reversion of the sensory input, has been extensively observed for many visual, auditory and somatosensory perceptual tasks (Williams et al., 1986; Kleinschmidt et al., 2002; Jackson et al., 2009). Similarly, the WTA neural network can produce hysteresis in perception if it is configured with multi-stable dynamics, but the spontaneous attractor is not essential (You et al., 2011). In addition, there are many other cognitive behaviors mediated by WTA competition, such as resource allocations of attention (Lee et al., 1999; Knudsen, 2007).

Synaptic excitation in neural networks is mainly mediated by AMPARs (α-amino-3-hydroxy-5-methyl-4-isoxazolepropionic acid receptors) and NMDARs. AMPAR-mediated synaptic currents exhibit sudden increases after a presynaptic spike and exponential decay with a short constant, whereas the NMDAR-mediated synaptic currents have a smooth rising phase and an exponential decay with a long time constant, thus leading to a saturation effect of synaptic currents (Wang, 1999). Experimental and theoretical studies have demonstrated that NMDARs play a significant role in WTA competition. NMDARs contribute not only to the slow time integration of sensory evidence in decision-making tasks (Wang, 2002; Wong and Wang, 2006) but also to the sustained activities during the delay observed in working memory tasks (Lisman et al., 1998).

Recently, several WTA neuromorphic circuits and hardware have implemented the above-mentioned cognitive functions, which benefits the design of elementary neuromorphic modules of many cognitive functions and the development of new generation of technologies that carry out brain-like artificial intelligence (Nere et al., 2012; Sandamirskaya, 2014) while maintaining remarkable energy efficiency (Indiveri and Horiuchi, 2011). For example, forced two-choice decision tasks have been implemented in a custom mixed signal analog/digital neuromorphic chip containing an array of 58 analog leaky integrate-and-fire (LIF) neurons and programmable synapses with realistic dynamics (Corradi et al., 2015). The discrete recurrent network configured in this chip is composed of two excitatory and one inhibitory population of silicon neurons coupled with local excitation and global inhibition, thus leading hard WTA competition. In addition, these tasks, based on the continuous recurrent network with local nearest neighboring excitatory connectivity and global inhibition, have been achieved in the LIF stop-learning WTA chip with 124 excitatory and 4 inhibitory neurons (Neftci and Indiveri, 2010; You, 2014). For working memory, an on-chip network composed of two excitatory and one inhibitory population of LIF neurons has been found to reproduce persistent activities observed in electrophysiological experiments (Giulioni et al., 2011). With its architecture different from that of the discrete decision on-chip network, this network has only one excitatory population distinguished by strong synaptic self-excitation, which leads to bistable attractor dynamics underlying meta-stable states of high-firing memory activity and low-firing spontaneous activity. Similarly, the continuous recurrent on-chip network has also been constructed as state-holding elements through proper configuration (Chicca et al., 2014). For hysteresis in the perception, no emulation in neuromorphic networks has been reported.

However, the non-linear dynamics of the NMDAR-mediated synaptic current is not considered in these neuromorphic systems; instead, saturation characteristics of silicon neurons and AMPAR-mediated synaptic currents (Bartolozzi and Indiveri, 2007) with a long time constant are considered. Although the decision tasks and working memory tasks can be implemented in these neuromorphic systems, the range of neural coding by the firing activity will become larger if the non-linearity of the NMDARs itself is considered. Moreover, the handling of mismatch and noise in the silicon is significant in neuromorphic systems. The non-linearity of the NMDARs increases the stability of attractor dynamics, especially the slow dynamics of ramp-up activities relative to the evidence integration in decision making and the stable dynamics of self-sustained activities in working memory.

In this work, we developed a WTA circuit with NMDARs by applying the dynamical system approach of circuit synthesis according to the two-variable version of a plausible biophysical WTA model, which was originally used for decision-making (Wong and Wang, 2006; Wong et al., 2007), and for working memory and hysteresis (You et al., 2011). In particular, we provided a step-by-step demonstration of how to build the NMDAR gating variable circuit, which is described by the first-order kinetic equation of a reversible chemical reaction. This circuit captured the non-linear dynamical characteristics of NMDAR gating variables. We simulated the neuromorphic WTA circuit for three WTA-related cognitive functions (decision-making, working memory and hysteresis in visual perception) in Cadence while analysing the WTA model through dynamical system approaches for corresponding cognitive functions. We compared the results between circuit simulations and theoretical analysis to investigate (1) whether the WTA circuit could implement decision tasks and reproduce gradually ramping neural activities observed in the electrophysiological recording in monkey experiments (Shadlen and Newsome, 2001) as the attractor dynamics of the WTA model predicts, (2) the sustained activities and the limited memory capacity in the WTA circuit during working memory and corresponding dynamics in the WTA model, and (3) whether the WTA circuit could achieve the hysteresis observed in the visual perception and operate according to the theoretical analysis for the WTA model.

Some of these results, such as circuit simulations for decision-making and corresponding theoretical analysis, have been presented in a preliminary form (You and Wang, 2016). In the current work, we more comprehensively demonstrate that the WTA circuit that we built has attractor dynamics underlying WTA-related cognitive functions, including decision-making, working memory and hysteresis behavior.

## Materials and methods

**Figure 1:**
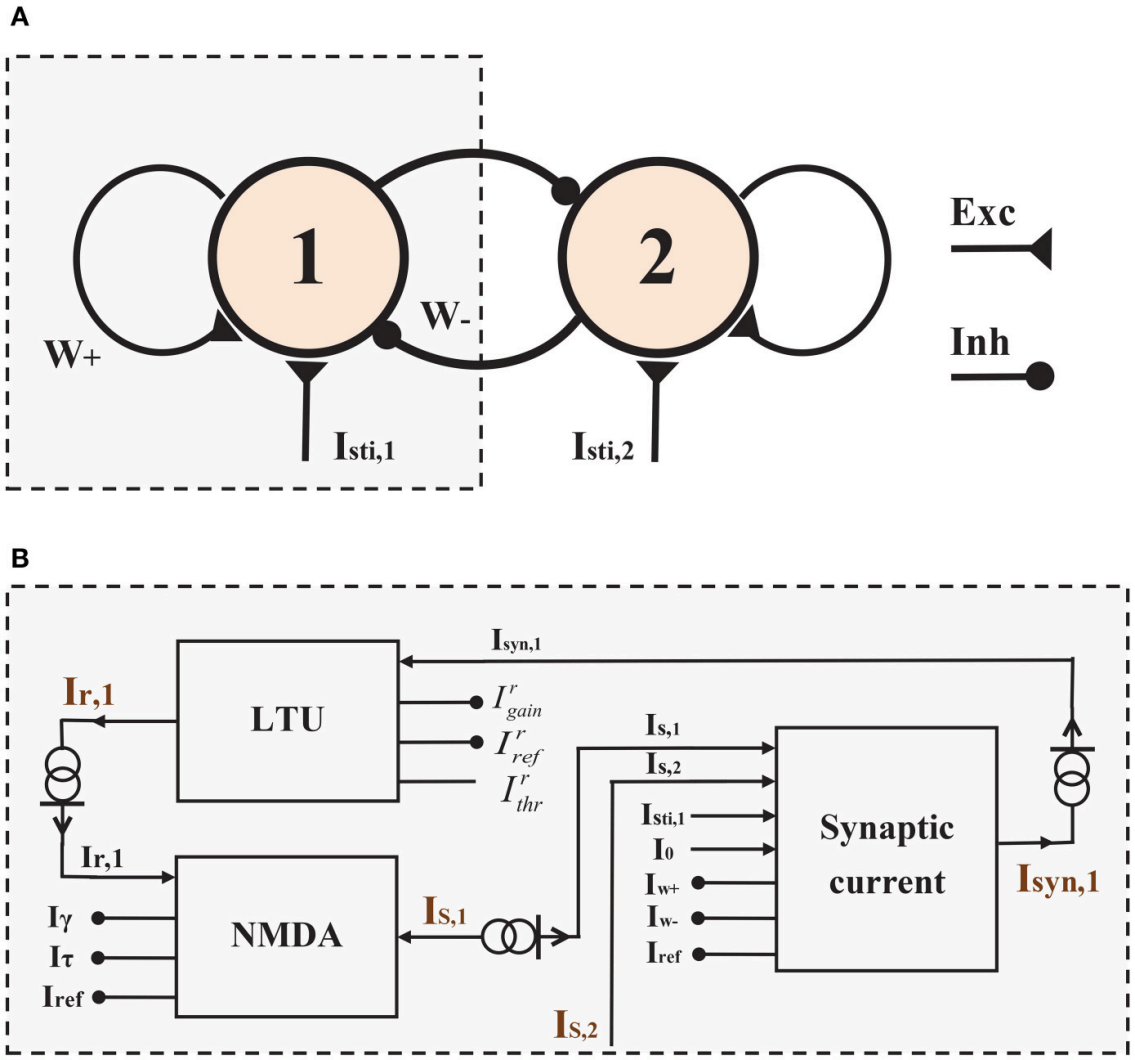
**Architecture of the Two-variable WTA Circuit**. **(A)** The two-variable WTA circuit consists of two neural populations endowed with self-excitation and effective mutual inhibition. **(B)** The block representation of the left half of the WTA circuit in **(A)**.

The neuromorphic WTA circuit designed herein was derived from a two-variable version of a biophysically realistic cortical model elucidating the cellular and circuit basis of decision making (Wong and Wang, 2006). In the previously reported reduced model, excitatory reverberation primarily mediated by NMDA receptors leads to the slow time integration of the motion evidence, and two dynamical variables *S*_1_ and *S*_2_ represent averaged gating variables of two populations selective for rightward and leftward motion directions. Our neuromorphic WTA circuit is composed of two neural populations with different preferences in some neural presentations (Figure 1A), such as two directions of the saccade in the decision task, and two spatial locations in the working memory task. In addition to external synaptic inputs *I*_*sti, i*_(*i* = 1, 2), two populations receive recurrent synaptic inputs including the self-excitation input with the synaptic weight *W*_+_ and the mutual-inhibition input with the synaptic weight *W*_−_. Experimental and theoretical research has found that the NMDA receptors at recurrent synapses are important to slow time integration in decision-making and persistent activities in working memory (Wong and Wang, 2006). Therefore, the recurrent excitation in our circuit is mediated by NMDARs, but could also be mediated by AMPARs if configured properly.

For the sake of simplicity, we show only the neuromorphic implementation of half of this WTA circuit in Figure 1B because of the symmetry of the circuit. At the circuit level, the circuit consists of three components: a linear-threshold unit circuit (LTU), an NMDA synapse circuit (NMDA) and a synaptic interaction circuit (Synaptic current). The LTU circuit realizes the activation function of a neural population (*I*_*r, i*_, *i* = 1, 2) with its total synaptic input (*I*_*syn, i*_). The NMDA synapse circuit produces the gating variable of the NMDARs (*S*_*i*_ = *I*_*S, i*_/*I*_*ref*_) according to the neural activity of each population (*I*_*r, i*_). The synaptic interaction circuit achieves the functionality of the total synaptic input (*I*_*syn, i*_) from self-excitation and mutual-inhibition (*I*_*S*, 1_ and *I*_*S*, 2_), and external sensory inputs (*I*_*sti, i*_). Details of these neuromorphic implementations are shown in the following parts of this section.

### Neuromorphic circuit of NMDAR gating variable

The dynamics of the NMDAR gating variable is characterized by a fast rise followed by a slow decay. When the presynaptic inputs at a recurrent synapse in a neural population are described by a Poisson spike train at a rate of *r* per cell, the slow dynamics of the average NMDAR gating variable *S* is characterized by the non-linear differential equation as follows (Wong and Wang, 2006):

(1) $$\begin{array}{l} \frac{dS}{dt}=-\frac{S}{\tau }+\left(1-S\right)\gamma r \end{array}$$

where τ is the decay time constant, and γ is a gain coefficient. To implement the circuit of the NMDAR gating variable, the corresponding mathematical expression is approximately transformed into the following current-mode description:

(2) $$\begin{array}{l} CU_{T}\frac{d}{dt}\frac{I_{S}}{I_{ref}}=-I_{\tau }\frac{I_{S}}{I_{ref}}+\left(1-\frac{I_{S}}{I_{ref}}\right)\frac{I_{\gamma }}{I_{ref}}I_{r} \end{array}$$

where dimensionless variables *S* and γ are replaced by two ratios of currents $\frac{I_{S}}{I_{ref}}$ and $\frac{I_{\gamma }}{I_{ref}}$, respectively. Because of the current-mode design, we use a current *I*_*r*_ to represent “the firing rate” *r* in this work, and we call *I*_*r*_ the neural activity in the remainder of this paper. The time constant $\tau =\frac{CU_{T}}{I_{\tau }}$.

**Figure 2:**
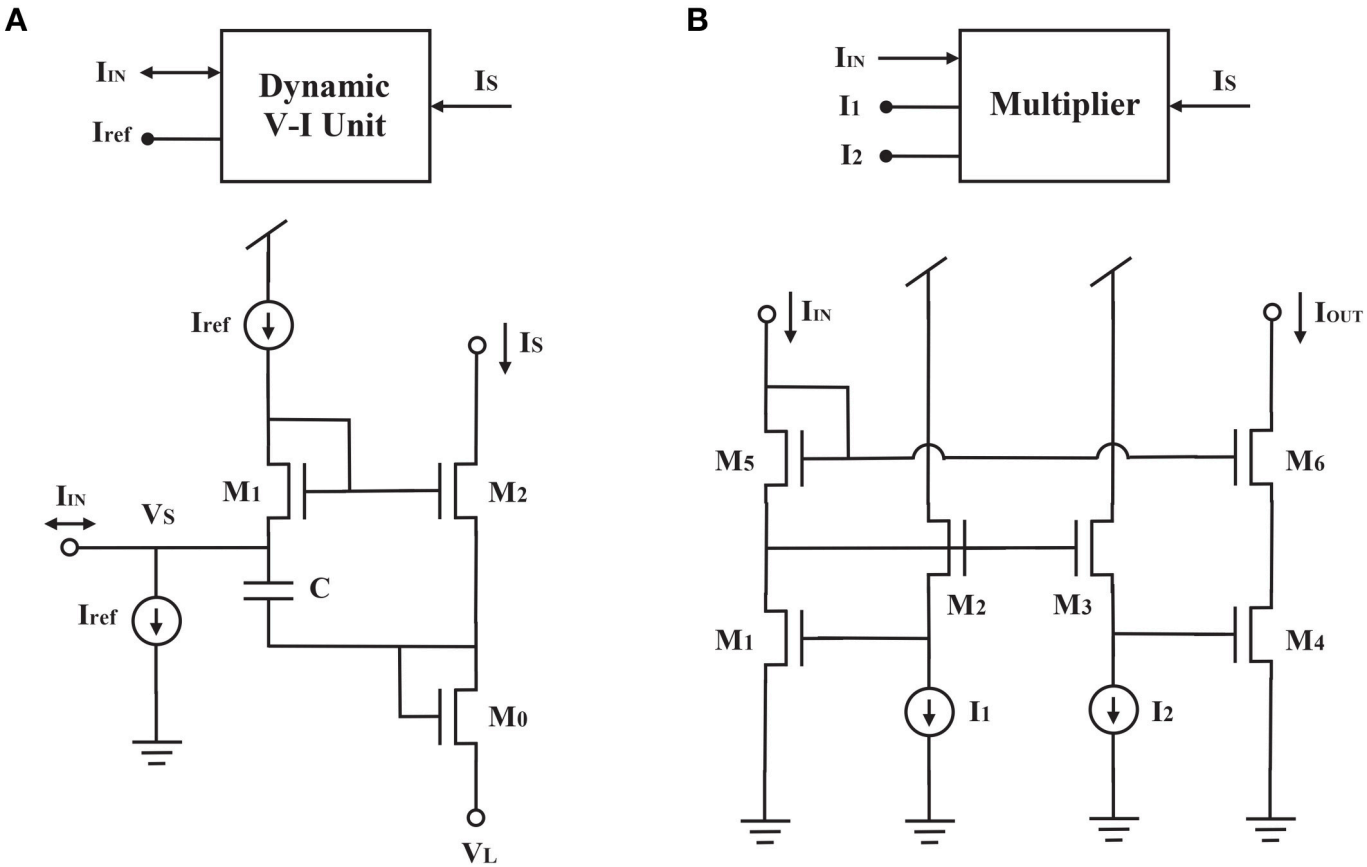
**Schematics of (A)** dynamic voltage-current unit (DVI) and **(B)** multiplier (M).

We used a dynamic voltage-current circuit (DVI) originally proposed for the log-domain circuit (Yu and Cauwenberghs, 2010) to achieve the derivative on the left side of Equation (2) (Figure 2A). Since

(3) $$\begin{array}{l} \frac{I_{S}}{I_{ref}}=\frac{I_{M_{2}}}{I_{M_{1}}}=e^{\frac{V_{S}-V_{0}}{U_{T}}} \end{array}$$

the dynamics of the voltage difference *V*_*S*_ − *V*_0_ has the following relationship with *I*_*S*_/*I*_*ref*_:

(4) $$\begin{array}{l} \frac{d}{dt}\frac{I_{S}}{I_{ref}}=\frac{1}{U_{T}}\frac{I_{S}}{I_{ref}}\frac{d}{dt}\left(V_{S}-V_{0}\right) \end{array}$$

By combining Equation (4) and Equation (2), we obtain the following voltage-mode differential equation to characterize the dynamics of *S*:

(5) $$\begin{array}{l} C\frac{d}{dt}\left(V_{S}-V_{0}\right)=-I_{\tau }+\frac{I_{\gamma }}{I_{S}}I_{r}-\frac{I_{\gamma }}{I_{ref}}I_{r} \end{array}$$

**Figure 3:**
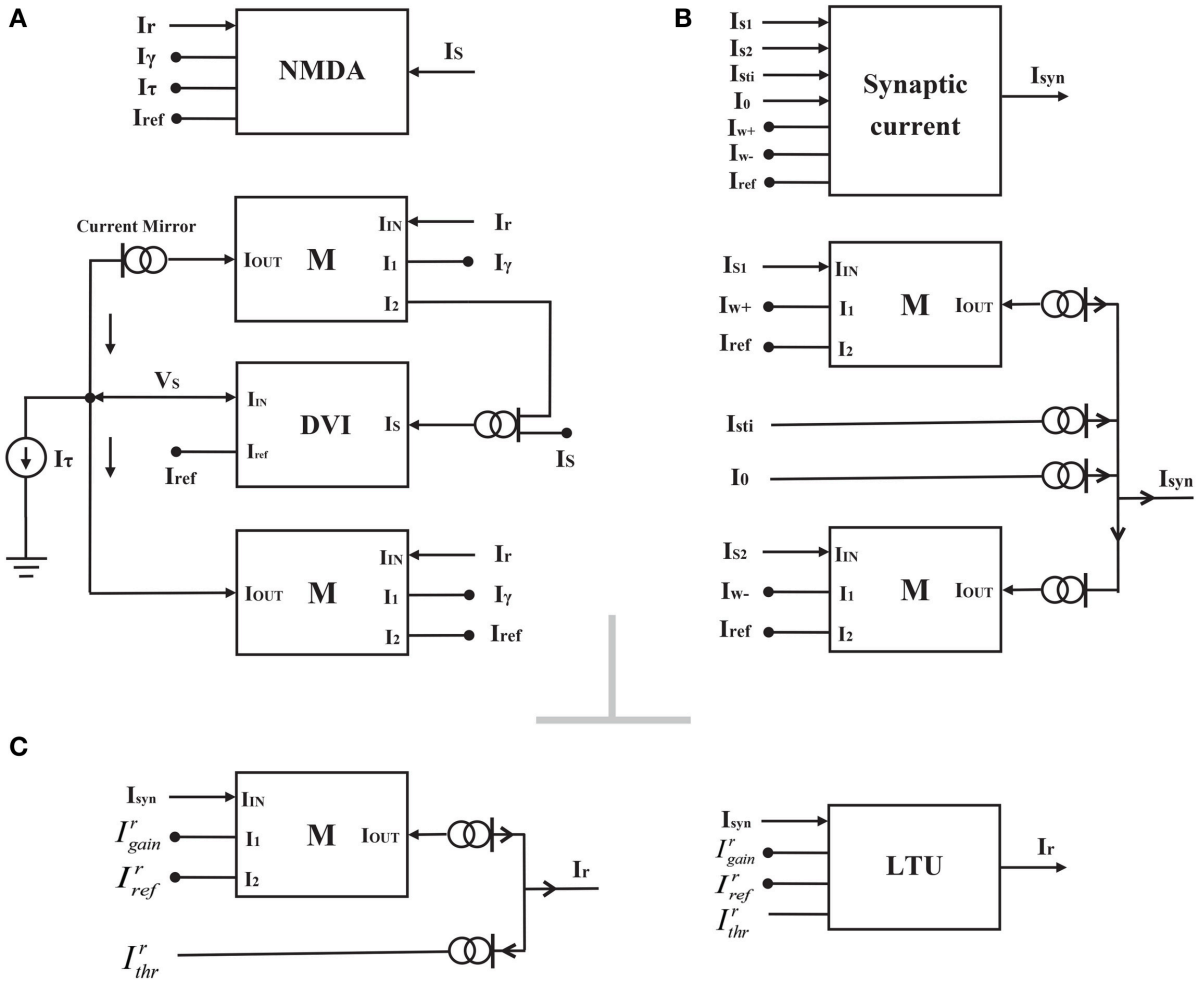
**The block representations of the NMDAR gating variable circuit (A)**, the synaptic current circuit **(B)** and the linear threshold unit circuit **(C)**.

According to the dynamical systems approach of circuit synthesis (Arthur and Boahen, 2011), three currents are required to drive the capacitor in the dynamic voltage-current circuit (Figures 2A, 3A): two currents decrease the capacitor voltage and one increases it. The first current term *I*_τ_ corresponds to the time constant of the circuit. The smaller its value is, the longer the time constant. The second term equals the product of the gain current *I*_γ_ and the activity current *I*_*r*_, divided by the gating variable current *I*_*S*_. This current is generated by a multiplier (Figures 2, 3) on the basis of the translinear principle for subthreshold transistors (Gilbert, 1975; Papadimitriou et al., 2013). It indicates that the driving force toward *V*_*DD*_ decreases with increasing *I*_*S*_. The third term corresponds to the driving force toward ground, and can also be generated by a multiplier (Figure 3A). Because both the second and third terms have the product of *I*_γ_ and *I*_*r*_, their multiplier circuits can share a common left branch of the multiplier in Figure 2B.

We concentrated on the steady state and the time constant for a given configuration as major dynamic characteristics of this circuit. From Equation (2), we obtain the steady state $\bar{S}$ and the time constant τ as follows:

(6) $$\begin{array}{l} \bar{S}=\frac{\frac{I_{\gamma }}{I_{ref}}I_{r}}{I_{\tau }+\frac{I_{\gamma }}{I_{ref}}I_{r}},\tau =\frac{CU_{T}}{I_{\tau }} \end{array}$$

Circuit simulations in Cadence in our previous work (You and Wang, 2016) have demonstrated that the NMDAR gating variable circuit can implement the major dynamics of its biological counterpart characterized by Equation (6): $\bar{S}$ increases with increasing *I*_*r*_ and *I*_γ_ but decreases with increasing *I*_τ_; the time constant of the circuit τ decreases with increasing *I*_τ_ but is independent of *I*_γ_ and *I*_*r*_.

### Neuromorphic implementation for a reduced two-variable WTA model

For the sake of neuromorphic implementations, the original reduced two-variable decision model (Wong and Wang, 2006) is re-expressed in the current-mode description as follows:

(7) $$\begin{array}{l} CU_{T}\frac{d}{dt}\frac{I_{S,i}}{I_{ref}}=-I_{\tau }\frac{I_{S,i}}{I_{ref}}+\left(1-\frac{I_{S,i}}{I_{ref}}\right)\frac{I_{\gamma }}{I_{ref}}I_{r,i} \end{array}$$

where *i* (= 1, 2) labels two selective populations. For simplicity, we adopted the linear-threshold unit (LTU) to model the neural activities of two populations *I*_*r, i*_, which are given by the equation as follows:

(8) $$\begin{array}{l} I_{r,i}={\left[\frac{I_{gain}^{r}}{I_{ref}^{r}}I_{syn,i}-I_{thr}^{r}\right]}^{+} \end{array}$$

where the dimensionless variable $\frac{I_{gain}^{r}}{I_{ref}^{r}}$ is the gain of the neural population, and $\frac{I_{ref}^{r}}{I_{gain}^{r}}I_{thr}^{r}$ is the threshold current. *I*_*syn, i*_ denotes the corresponding synaptic current. [*x*]^+^ is equivalent to the function *max*(*x*, 0). According to Equation (8), we require only a multiplier and several current mirrors to realize the LTU circuit (Figure 3C). However, the neural activity *I*_*r*_ of this LTU circuit in circuit simulations increases smoothly, not suddenly, as described by Equation (8) when the synaptic current *I*_*syn*_ approaches the threshold current. Therefore, we chose a new activation model instead of Equation (8) in the following system approach analysis:

(9) $$\begin{array}{l} I_{r,i}=\frac{\frac{I_{gain}^{r}}{I_{ref}^{r}}I_{syn,i}-I_{thr}^{r}}{1-\exp\left[-g\left(\frac{I_{gain}^{r}}{I_{ref}^{r}}I_{syn,i}-I_{thr}^{r}\right)\right]} \end{array}$$

where the parameter *g* tunes the smoothness of the activation curve around the threshold current. Equation (9) is more biophysically realistic for the response of neural populations.

The total synaptic currents of two selective populations *I*_*syn, i*_ are given by the following equations:

(10) $$\left\{ \begin{array}{l} I_{syn,1}=I_{w^{+}}\frac{I_{S,1}}{I_{ref}}-I_{w^{-}}\frac{I_{S,2}}{I_{ref}}+I_{0}+I_{sti,1}\\ I_{syn,2}=I_{w^{+}}\frac{I_{S,2}}{I_{ref}}-I_{w^{-}}\frac{I_{S,1}}{I_{ref}}+I_{0}+I_{sti,2} \end{array}\right.$$

where *I*_*sti, i*_ represents the external sensory stimulus to the population *i*, and *I*_0_ is the effective background input. $I_{w^{+}}$ and $I_{w^{-}}$ are the effective self-excitation and mutual-inhibition currents per unit (the gating variable) in the recurrent network, respectively. The synaptic current block can also be realized by two multipliers and several current mirrors (Figure 3B). However, for this synaptic current circuit shown in Figure 3B, *I*_*syn*_ is close to but still larger than 0 when *I*_*s*1_ < *I*_*s*2_, indicating that our synaptic current circuit looks like a rectifier ([·]^+^). Although this result is not consistent with the response determined by Equation (10), it has little influence on the implementation of attractor dynamics in the decision circuit, owing to the rectifying effect of the LTU circuit.

We built the neuromorphic circuit of this two-variable WTA model on the basis of Equation (7), (8), and (10). Figure 1B shows the block representation of the WTA circuit.

### Circuit configurations of WTA-related cognitive functions

Configuring parameters of the simplified two-variable WTA model described by Equations (7–10) allows implementation of decision-making, working memory and hysteresis in visual perception (Wong and Wang, 2006; You et al., 2011). In this work, to test the responses of the neuromorphic WTA circuit built in Cadence (Figure 1), we configured the circuit and design simulation protocols for three cognitive tasks. Bias currents configured in the circuit are as follows: *I*_τ_ = 5*pA*, *I*_γ_ = 10*pA*, *I*_*ref*_ = 100*pA*, $I_{gain}^{r}=100pA$, $I_{thr}^{r}=50pA$, $I_{ref}^{r}=100pA$, *I*_0_ = 15*pA*. The capacitance of the capacitors used in the circuit of the NMDAR gating variable *C* = 20*pF*. The parameter *g* = 0.2 from simulations. We also used the above values as parameters in the model for theoretical analysis. Furthermore, *U*_*T*_ = 25*mV* in the model. Bias currents $I_{w^{+}}$ and $I_{w^{-}}$ are task specific.

#### Decision-making

**Figure 4:**
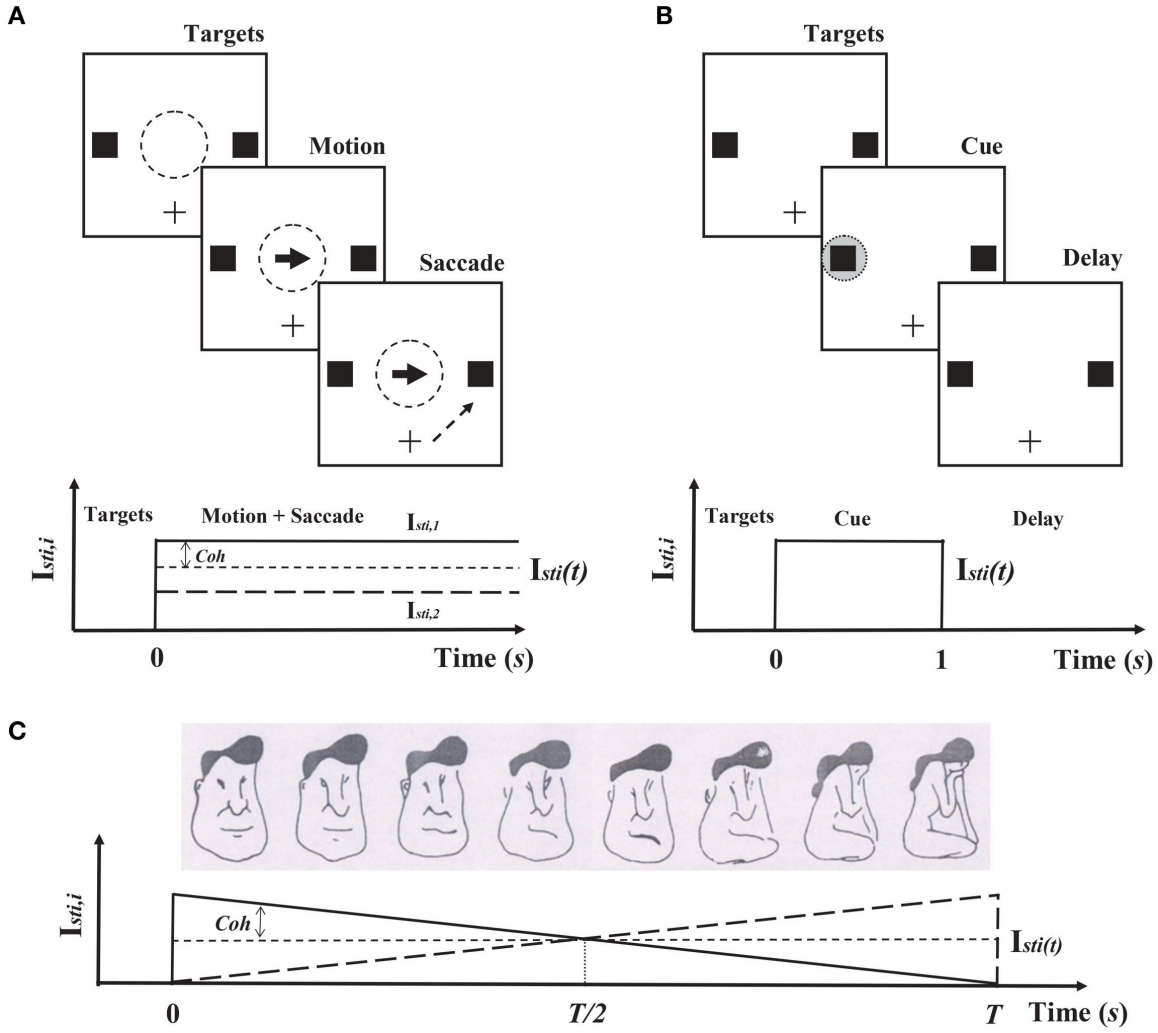
**Cognitive tasks and corresponding circuit simulation protocols in Cadence**. **(A)** Decision-making. In this task, the subject is required to fixate on the center of the monitor, and two peripheral targets are presented (Top). After a delay, a fraction of dots move coherently toward one of the targets, while the remaining dots move randomly. The subject is required to report the direction of the coherent motion by a saccade to one of the targets. The difficulty of this task is determined by the fraction of coherently moving dots (the coherence level: Coh). In the circuit simulation in Cadence, the motion stimulus is presented during the motion and saccade stages (Bottom). **(B)** Working memory. In this task, one or two gray circles as cues are presented during the cue stage, and the subject is required to recall the locations of the cues during the delay stage. **(C)** Hysteresis in visual perception. In this task, the figures from left to right morph from a man's face to a kneeling girl, which is adapted from Haken (1983) and Chialvo and Apkarian (1993). The subject is required to report what was perceived. In both sequence trials (left ↔ right), a typical hysteresis loop of the subject's perception is shown. Because the features of the man's face and the kneeling girl change smoothly and in a reverse manner, the sensory stimulus in the Cadence simulation is given in the same way during the presentation of the figures (Bottom).

In discrimination tasks of coherent motion in random dots, neurophysiologists have found that the neural activities in monkeys' lateral intraparietal cortexes (LIP) are related to neural computation of decision-making (Shadlen and Newsome, 2001; Shadlen and Kiani, 2013). This two-variable WTA model has been proposed to explain the underlying mechanism of decisions in LIP (Wang, 2002; Wong and Wang, 2006). Therefore, two populations in the neuromorphic WTA circuit are set to prefer two alternative directions of coherently moving dots. According to this discrimination task (Figure 4A), the external sensory stimuli (moving dots) to two populations are

(11) $$\{\begin{array}{c} I_{sti,1}=I_{sti}(t)(1+Coh)\\ I_{sti,2}=I_{sti}(t)(1-Coh) \end{array}$$

where the input strength of the stimuli *I*_*sti*_(*t*) is 0*pA* for *t* < 0 and 15*pA* for *t* ≥ 0. The coherence level *Coh* represents the percentage of coherently moving dots toward one direction. After the stimulus onset, the circuit is required to make its decision by gradually ramping activity of either population.

#### Working memory

Neurophysiological experiments on monkeys have reported that persistent activities during delayed response tasks maintain working memory information (Compte et al., 2003). Mnemonic activity is thought to be sustained by synaptic reverberation in a recurrent circuit, and its stability is achieved mainly by NMDAR-mediated reverberation (Wang, 2001). Therefore, the WTA model (Equation 7–10) endowed with NMDAR-mediated reverberation has the ability of working memory. According to the procedure of the delay response task (Figure 4B), one or two gray circles are presented as the sensory stimuli to two populations. Thus, during the cue presentation, either *I*_*sti*, 1_ or *I*_*sti*, 2_ is 70*pA* and the other is 0*pA* when the memory load is one, and both of them are 50*pA* when the memory load is two. *I*_*sti*, 1_ and *I*_*sti*, 2_ are also 0*pA* before the cue stimuli onset and after the cue stimuli offset.

#### Hysteresis in visual perception

Hysteresis is a typical phenomenon that depends on previous experiences in visual, auditory and somatosensory perceptions (Williams et al., 1986; Kleinschmidt et al., 2002; Jackson et al., 2009). Theoretical analysis has revealed that a neural model with a similar structure to our neuromorphic circuit can implement hysteresis in visual perception (You et al., 2011). Here, we chose the discrimination task between the man's face and the kneeling girl as the prototype for our circuit simulation in Cadence (Figure 4C). The first population in our circuit represented the man's face, and the other represented the kneeling girl. According to this task, the sensory stimulus was presented only from 0*s* to *Ts*, and the figure changed from the man's face to the kneeling girl. Therefore, we used the similar simulation protocol shown in Equation (11), but with the constant *I*_*sti*_(*t*) and varying coherence level *Coh* over time. *I*_*sti*_(*t*) is 15*pA* and *Coh* = 1 − 2*t*/*T*. *T* = 20*s*.

## Results

### Emulation of decisions and their attractor dynamics in the neuromorphic WTA circuit with NMDARs

**Figure 5:**
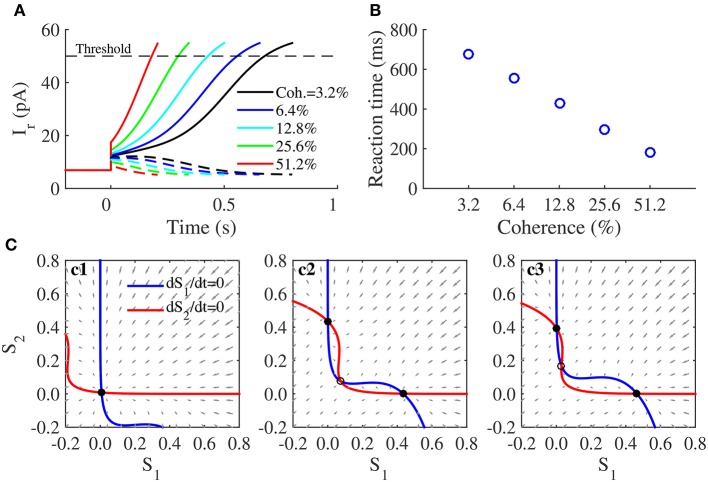
**Decision-making and its attractor dynamics in the neuromorphic WTA circuit**. **(A)** Firing activities in the decision task. The stimuli are presented to the circuit after 0*s*. Colors indicate different coherence levels of the stimuli. $I_{w^{+}}=100pA$, and $I_{w^{-}}=60pA$. **(B)** The decision time decreases as the coherence level increases. **(C)** Phase-plane plot for two neural populations in the decision task, without external input (**c1**), in the presence of unbiased stimuli (**c2**:*Coh*. = 0%) or biased stimuli (**c3**:*Coh*. = 12.8%). Solid dots denote stable steady states, and circles denote unstable steady states. $I_{w^{+}}=100pA$, and $I_{w^{-}}=60pA$. The negative regions in these phase-plane plots do not make sense biologically but are beneficial for showing attractor dynamics in the neuromorphic WTA circuit clearly.

We tested the responses of this decision circuit through simulations given the stimulus with different coherence levels, which were given after 0*s* (Figure 5A). Responses of both populations stay at a low activity before the stimulus onset. After the stimulus was presented, one of them exhibited a gradual ramp-up activity, and the other exhibited a gradually decreasing activity. According to the decision bound theory supported by some observations in the monkey experiment (Kiani et al., 2008), we set a fixed neural activity (corresponding to a fixed *I*_*r*_ 50*pA*) as the decision threshold in our circuit simulations. A decision is made once the activity of any population exceeds the decision threshold, and corresponding time is measured as the reaction time. The speed of the ramp-up activity increased with increasing coherence of the stimulus, whereas the reaction time decreased (Figure 5B); both these observations are consistent with neural activities and behaviors observed with the monkey experiments (Shadlen and Newsome, 2001). However, because our decision circuit is a noise-free system, the reaction time had a linear relationship with the logarithm of the coherence level (Figure 5B), as has been observed in previous studies (You, 2014).

These simulation results are consistent with results from a phase-plane analysis of the model described by Equation (7), (8), and (10) (Figure 5C). In the (*S*_1_,*S*_2_) phase space (decision space), two lines called nullclines are plotted first by setting the dynamical equations *dS*_1_/*dt* = 0 and *dS*_2_/*dt* = 0 (Equation 7). The intersections of two nullclines are steady states of the model. The direction field in the phase space shows how the system state will evolve. In the absence of a stimulus, the two nullclines intersected with each other only once, thus producing a stable steady state, that is, they function as an attractor (Figure 5c1), thus explaining why the two populations in the decision circuit had low spontaneous activities before the stimulus onset. When an unbiased stimulus was presented (*Coh*. = 0%), two nullclines intersected with each other three times and produced a saddle-type unstable and two stable steady states (Figure 5c2). They formed two basins of attraction around different two attractors, representing two alternatives in the decision. The system state was located in the vicinity of the unstable steady state before the stimulus onset and then evolved toward one of two attractors after the stimulus onset. This saddle node structure determined the time course of neural activities underlying the two-alternative decision. When a biased stimulus was applied, the phase space was no longer symmetrical. As an example of the biased stimulus *Coh*. = 12.8% (Figure 5c3), the unstable steady state was closer to the attractor representing the second alternative, thus indicating that the attractor state responsible for the first alternative has a larger basin of attraction than the other. After the onset of a biased stimulus, the initial state of the system already lies within the basin of the attractor of the first alternative, and the system state will evolve toward its favored attractor, especially in our noise-free circuit.

### The dynamics of the circuit is consistent with the bifurcation analysis of the WTA model in the decision task

**Figure 6:**
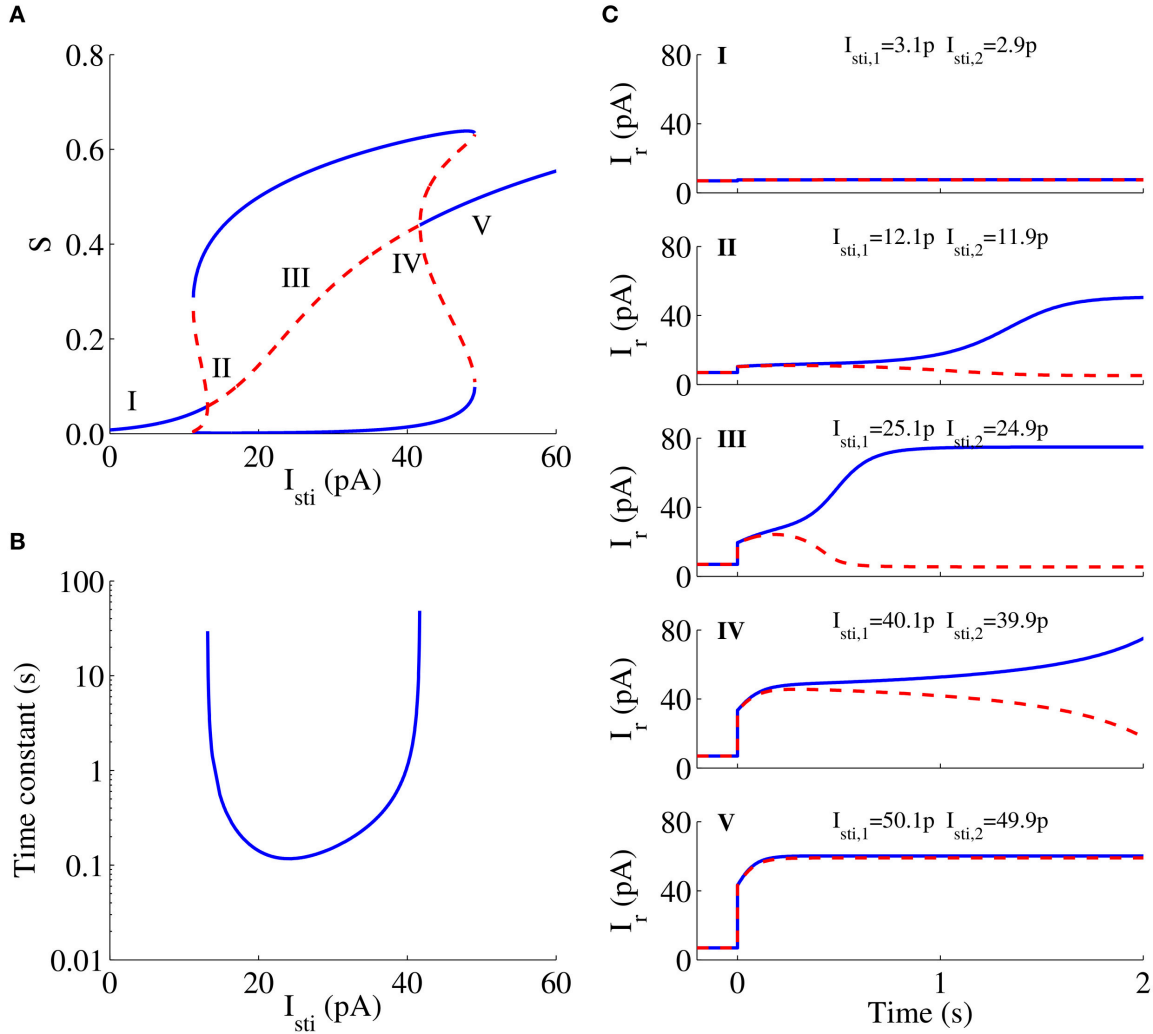
**Bifurcation diagram of a neural population with the unbiased stimulus *I*_*sti*_ and neural responses to different *I*_*sti*_ in the neuromorphic WTA circuit**. **(A)** Bifurcation diagram. Bold lines, stable steady states; dashed lines, unstable steady states. **(B)** Time constant. **(C)** Neural responses to different stimuli *I*_*sti*_ are consistent with expectations in the bifurcation analysis in the WTA circuit.

Figure 6A shows the bifurcation diagram of a neural population with the unbiased external stimulus *I*_*sti*_ as a parameter in the WTA model for the decision task and presents the relationship between the steady states of the system and the stimulus strength. In this bifurcation diagram, the gradually increasing middle curve represents symmetric steady states, and the upper and lower curves represent asymmetric states. Blue solid curves and red dashed curves denote stable and unstable steady states, respectively. The neuromorphic WTA circuit shows spontaneous activities at a low rate when the stimulus is small (Figure 6CI) in the decision task, because there is only one stable steady state for the weak stimulus (the region *I* in Figure 6A). As the external stimulus increases further, the system has three stable steady states (two asymmetric and one symmetric) and two unstable steady states (asymmetric). In this case, the system shows stronger spontaneous activities. For the stronger external stimulus, however, the system will develop one unstable symmetric steady state and two stable asymmetric steady states in the phase space (regions II–IV in Figure 6A), thus leading to WTA behavior (Figures 6CII–IV). The time constant of the unstable manifold of this symmetric state is very large and decreases with increasing stimulus strength for low strength in this region, but increases again for high strength (Figure 6B). The simulation results of the circuit were also consistent with the prediction of its bifurcation analysis for the WTA model. The divergence speed of neural activities for the stimulus strength around regions *II* and *IV* is larger than that around region *III* (Figures 6CII–IV). When the stimulus strength further increases, and the symmetric steady state becomes stable again, the system has another two asymmetric stable steady states and two asymmetric unstable steady states (Figure 6A). If the stimulus strength is too large, the system has the sole steady state that is stable. The system cannot develop the ramping-up activity and make a decision (Figure 6CV).

### Emulation of working memory and its attractor dynamics in the neuromorphic WTA circuit

We concentrated on following aspects of working memory when we performed this cognitive task in the neuromorphic WTA circuit: the ability of working memory and the limitation of its capacity. In circuit simulations, we verified the working memory capacity limit under two circuit configurations ($I_{w^{+}}=240pA$ and 300*pA*). We also applied the stimuli with one or two items to the circuit during the cue presentation according to the protocol of working memory (Figure 4B).

**Figure 7:**
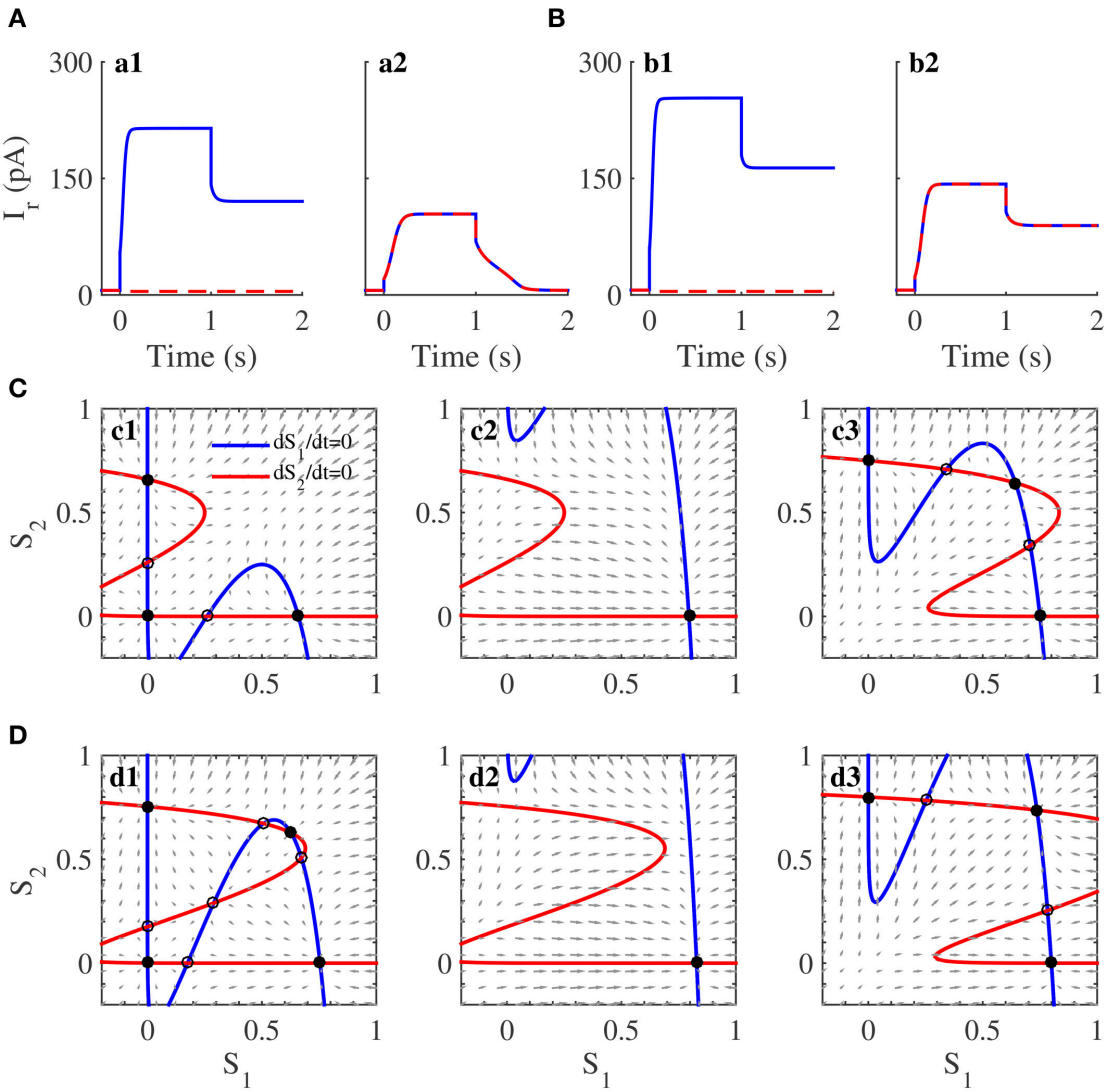
**Working memory capacity and its attractor dynamics in the neuromorphic WTA circuit**. **(A)** Neural activities of two neural populations in the WTA circuit with the self-excitation strength $I_{w^{+}}=240pA$. Persistent activity shows after the cancellation of the stimulus for one item but not two items. **(B)** Neural activation plot when $I_{w^{+}}=300pA$. Persistent activities show during the delay for both one item and two items. **(C,D)** Phase-plane plot for two neural populations in working memory tasks without external input (**c1** and **d1**), in the presence of one item (**c2** and **d2**) and two items (**c3** and **d3**). Because of unprecise mapping of the WTA model onto the WTA circuit, we set $I_{w^{+}}$ as 200*pA* in **(C)** and 250*pA* in **(D)**, which correspond to those in **(A,B)**.

The neural activity patterns of the circuit with $I_{w^{+}}=240pA$ in the circuit simulations are shown in Figure 7A. Several characteristics are worth noting. First, two neural populations were spontaneously active at a low rate before the onset of the cue stimuli. Second, the neural populations, which encoded the directions of the items that appeared during the cue period, increased their neural activities. When only one item was presented, only the corresponding neural population fired (Figure 7a1). When two items appeared, both neural populations developed strong activities (Figure 7a2). Third, the strong activity persisted throughout the delay period when the working memory load comprised one item (Figure 7a1), but the strong activities decayed gradually when the load size was two (Figure 7a2). The results from the above circuit simulations revealed that the WTA circuit with $I_{w^{+}}=240pA$ could remember only one item.

The results from the phase-plane analysis for the WTA model were also able to account for these observations (Figure 7C). For the system without the cue presentation (Figure 7c1), two nullclines intersected with each other five times, thus producing three stable steady states and two unstable steady states. These three stable steady states, around which three basins of attraction formed, were divided into two classes: one symmetric low state, called the spontaneous state, and two asymmetric high states, called memory states. The WTA circuit stayed at this spontaneous state before the cue presentation. After the onset of the cue, if the memory load was one item, the system had only one attractor (Figure 7c2), and then one population in the WTA circuit evolved from its spontaneous state to the high activity (Figure 7a1). If the memory load was two items, the system had three attractors (Figure 7c3). Because the initial state of the circuit was located in the attraction basin of the attractor with two high values, the activities of both populations increased (Figure 7a2). After the cancellation of the cue, the system evolved from the attractor in Figure 7c2 to its closest memory state (the south-east asymmetric attractor) in Figure 7c1 for one item, but from the attractor with two high values in Figure 7c3 to the spontaneous state in Figure 7c1 for two items. These results illustrate why the WTA circuit could hold only one item in its working memory.

When $I_{w^{+}}$ was configured to be 300*pA*, all populations subjected to the cue items, showed persistent activities after the cue stimuli offset (Figure 7B). The memory capacity was two in this configuration, which was also illustrated by the similar phase-plane analysis for the WTA model. Given the cue stimuli with one item and two items, the dynamics of the system characterized by Figures 7d2,d3 was similar to those of Figures 7c2,c3. Therefore, the WTA circuit behaved similarly during the cue presentation for both configurations of recurrent excitatory connection strength (Figures 7A,B). However, two nullclines intersected nine times, producing four basins of attraction, among which there was a memory attractor with two high values representing the memory of two items (Figure 7d1). In the case of the stimuli with two items, the system state did not evolve toward the spontaneous state but instead evolved toward its closest memory state responsible for the memory of two items. Thus, the WTA circuit was able to hold persistent activities during the delay and remember two items (Figure 7b2).

### Hysteresis in visual perception

**Figure 8:**
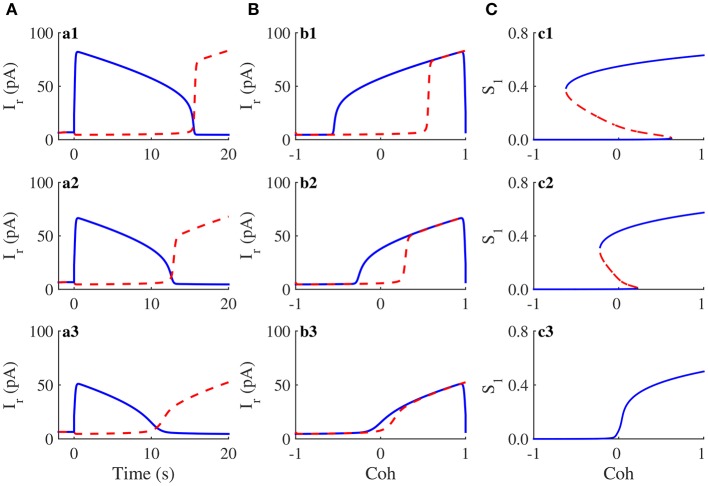
**Hysteresis in visual perception and its attractor dynamics in the neuromorphic WTA circuit**. **(A)** The time course of activities of two neural populations when $I_{w^{+}}$ is 100*pA* (**a1**), 80*pA* (**a2**), and 60*pA* (**a3**), respectively. **(B)** The activity of the man's face selective population exhibits hysteresis. The sequence of $I_{w^{+}}$ is the same as that in **(A)**. The hysteresis effect decreases with decreasing $I_{w^{+}}$, and there is no hysteresis when $I_{w^{+}}$ is small enough. **(C)** Bifurcation diagram of a selective population with the feature coherence of figures *Coh* as parameter. Bold lines, stable steady states; dashed lines, saddle steady states. Because the mapping of the WTA model onto the neuromorphic WTA circuit cannot be precise, $I_{w^{+}}$ is 120*pA*, 100*pA*, and 80*pA*, respectively.

Our previous work has revealed that hysteresis in visual perception can be reproduced in the simulation of the two-variable WTA model (You et al., 2011). In circuit simulations, we applied the stimulus protocol of the discrimination task between the man's face and the kneeling girl to the neuromorphic WTA circuit with three different $I_{w^{+}}$. Corresponding activity plots are shown in Figure 8A. In the circuit simulation for $I_{w^{+}}=100pA$ (Figure 8a1), the activity of the population representing the man's face (solid curve) gradually decreased and that of the kneeling girl (dashed curve) increases with the changing stimulus. Notably, the crossover of two activities (15.5*s*) was followed by the crossover of the stimulus to two populations (10*s*). Thus, the circuit perceives the kneeling girl later than the time at which its feature exceeds the feature of the man's face; that is, a hysteresis phenomenon in the visual perception was observed. The corresponding hysteresis loop of neural activity is shown in Figure 8. Similarly, a hysteresis phenomenon was also observed for $I_{w^{+}}=80pA$, but the corresponding hysteresis effect weakened (Figures 8a2,b2). If $I_{w^{+}}$ decreased further, for example, 60*pA*, the hysteresis effect disappeared (Figures 8a3,b3).

To compare the circuit simulation results and theoretical analysis results, we calculated the steady states of the WTA model with the variant parameter $I_{w^{+}}$ and the increasing feature coherence *Coh*. However, because of the unprecise mapping of the WTA model onto the neuromorphic WTA circuit, we chose another set of $I_{w^{+}}$s (120, 100, and 80*pA*) in the WTA model for comparisons. When $I_{w^{+}}=120pA$, the system had strong self-excitation. Along with the increase in *Coh*, the number of steady states of the system changed from one to three and then to one (Figure 8c1). There was a *Coh* interval around 0, in which the system had three steady states, including two stable and one unstable. These steady states accounted for the hysteresis in the visual perception (Figures 8a1,b1). If $I_{w^{+}}$ decreased and was equal to 100*pA*, the *Coh* interval with three steady states decreases (Figure 8c2), thus revealing a weakened hysteresis effect of the visual perception (Figures 8a2,b2). When $I_{w^{+}}=80pA$, the *Coh* interval with three steady states disappeared and the system had a single steady state (Figure 8c3). Thus, there was no hysteresis in the visual perception (Figures 8a3,b3). Although there was a slight mapping mismatch from the WTA model onto the neuromorphic WTA circuit, the above circuit simulation results and theoretical analysis results demonstrated that the neuromorphic WTA circuit mimics hysteresis behavior in the visual discrimination task.

## Conclusions and discussion

In this work, we designed a WTA circuit with NMDARs by using a dynamical system approach of circuit synthesis according to the two-variable version of a plausible biophysical WTA model (Wong and Wang, 2006; Arthur and Boahen, 2011). Comparing the results between circuit simulations of this neuromorphic WTA circuit and theoretical analysis of the current-mode WTA model, we demonstrated that the WTA circuit with NMDARs was able to implement attractor dynamics underlying cognitive functions, such as decision-making, working memory and hysteresis in visual perception. For decision tasks, the WTA circuit reproduced gradual ramp-up neural activities determined by the saddle node structure and the slow integration of sensory evidence. Furthermore, the divergence speed of neural activities was also consistent with the prediction of the time constants of corresponding saddle points. For working memory tasks, the WTA circuit showed sustained activities determined by multi-stable dynamics, that is, the spontaneous state and memory states coexisted. More interestingly, the memory capacity of the WTA circuit was able to be tuned by configuring the recurrent excitatory connection strength, as has been discussed in a continuous spiking network model (Wei et al., 2012). During discrimination tasks between a man's face and a kneeling girl, the WTA circuit reproduced the typical hysteresis behavior observed in visual, auditory and somatosensory perceptions (Williams et al., 1986; Kleinschmidt et al., 2002; Jackson et al., 2009) and predicted by bifurcation analysis with feature coherence *Coh* as a parameter. Furthermore, the hysteresis effect was able to be regulated by configuring the recurrent connection strength in the WTA circuit. It was also critical to implement the non-linear dynamics of the NMDAR-mediated synaptic current in the WTA circuit for the above cognitive tasks. These results demonstrated that our WTA circuit was able to implement attractor dynamics underlying WTA-related cognitive functions.

Quantitatively accurate neuromorphic mapping is important for neuromorphic systems to emulate computations carried out in the nervous system in VLSI hardware. In this study, we designed and implemented a neuromorphic WTA circuit by using a dynamical system design, which has been applied for mapping of quantitative neuronal models onto neuromorphic hardware (Takemoto and Kohno, 2007; Arthur and Boahen, 2011; Gao et al., 2012). However, imprecise mapping of the WTA model onto the WTA circuit is inevitable because of factors such as ideal modeling of transistors, inaccurate current replica. Parameters configured for circuit simulations of the WTA circuit in Cadence and theoretical analysis of the WTA model during three cognitive tasks are not same but are similar. Thus, the dynamical system approach of circuit synthesis in our work was effective. The circuit simulation results demonstrated that the WTA circuit produced behaviors that was predicted and explained by the WTA model, such as the divergence speed of neural activities predicted by the time constants of corresponding saddle points in implementing decision tasks and the working memory capacity determined by memory attractors in the phase-plane analysis when implementing working memory tasks. In addition, the mismatch is inevitable because identical devices suffer from random mismatch, which stems from microscopic fluctuations in dimensions, doping, oxide thickness and a host of other causes (Hastings, 2005; Sun and Basu, 2011). The mismatch can destroy the symmetry of the WTA circuit and deteriorate performance during cognitive tasks. For example, the probability of choosing either of two options will not be 50% for an unbiased stimulus (*Coh*. = 0%) during decision tasks. However, weakly biased behavior is acceptable if the asymmetry of the WTA circuit is not made severe by reducing its mismatch.

The primary components of the networks in the brain are neurons, which transfer information by digital means (the pulse, called a “spike” or “action potential”) and process information by analog means. Therefore, most neuromorphic systems are event based. Hence massively parallel, low-power, and inexpensive computing architectures are promising (Boahen, 2000; Liu et al., 2015), such as neuromorphic systems from the COLAMN project (Wijekoon and Dudek, 2012), NCS research project (Qiao et al., 2015), the Neurogrid project (Benjamin et al., 2014), the SpiNNaker project (Furber et al., 2014). Most silicon neurons on hardware are built according to various spike neuron models, such as leaky integrate-and-fire neurons (Indiveri et al., 2006; Gao et al., 2012), Hodgkin-Huxley neurons (Saighi et al., 2011), Izhikevich neurons (Mizoguchi et al., 2011) and other two-variable neurons (Takemoto et al., 2011). Non-spiking neurons, described by various activation transfer functions, are used in convolutional and deep neural networks on hardware (Krizhevsky et al., 2012; Chen et al., 2014; Luo et al., 2017). In this study, the two-variable WTA model is the firing rate model, not the spiking neuron model. Therefore, we developed the WTA circuit in a current-mode manner, and the information transfer in the WTA circuit operates in the analog mode. This methodology is practical because the WTA circuit is small-scale and does not have too much information transferred inside. These independent cognitive modules can be configured for some cognitive tasks and can be assembled to construct larger-scale neuromorphic systems; moreover, communications among the WTA circuits can be event based for high-efficiency and low-power design.

Using the relationship between dynamic field theory (DFT) and soft WTA networks, Sandamirskaya has systematically revised and integrated DFT mechanisms that may be implemented in neuromorphic devices to achieve working memory, intentionality or autonomous learning (Sandamirskaya, 2014). The main characterizations of the dynamic neural fields underlying the above cognitive functions are detection instability, selection instability, working memory instability and reverse detection instability. The WTA circuit can also present the above characterizations, which were demonstrated by circuit simulation and theoretical analysis results in this study. Moreover, the WTA circuit displays rich repertories of dynamics underlying cognitive behavior after being configured with NMDARs. Therefore, the WTA circuit as a basic cognitive module, together with other functions, such as associative memory (Hu et al., 2015) and synaptic plasticity (Fusi et al., 2000; Indiveri et al., 2010), may be extended and assembled into the framework of higher neuromophic cognitive systems. For instance, this WTA architecture, may be used as state-holding elements to be assembled into finite state machines (FSM) (Neftci et al., 2013), or as WTA elements to be assembled into a sophisticated classifier network (Shim et al., 2016).

Compared with continuous models of dynamic neural fields, the two-variable WTA circuit is based on the discrete model, and its neural coding ability determined by the state space is limited. However, theoretical analysis reveals that the WTA model with more than two populations also has similar dynamics to that discussed above (McMillen and Holmes, 2006; Albantakis and Deco, 2009). By using the dynamical system approach of circuit synthesis, the WTA circuit could be constructed for a given number of populations to implement more complex cognitive behavior with more alternatives, memory items or classifiers. However, the scale of the WTA circuit cannot be too large, because the complexity of synaptic interactions on hardware would grow according to n-squared with an increasing number of populations.

## Author contributions

HY, Designed the circuit, performed circuit simulations and theoretical analysis, and wrote the manuscript. DW, Helped in theoretical analysis, to evaluate and edit the manuscript.

### Conflict of interest statement

The authors declare that the research was conducted in the absence of any commercial or financial relationships that could be construed as a potential conflict of interest.
